# Sleeping Sickness in Travelers - Do They Really Sleep?

**DOI:** 10.1371/journal.pntd.0001358

**Published:** 2011-11-01

**Authors:** Karin Urech, Andreas Neumayr, Johannes Blum

**Affiliations:** 1 Swiss Tropical and Public Heath Institute, Basel, Switzerland; 2 Medical Faculty, University of Basel, Basel, Switzerland; University of Pittsburgh, United States of America

## Abstract

The number of imported Human African Trypanosomiasis (HAT) cases in non-endemic countries has increased over the last years. The objective of this analysis is to describe the clinical presentation of HAT in Caucasian travelers. Literature was screened (MEDLINE, Pubmed) using the terms “Human African Trypanosomiasis”, “travelers” and “expatriates”; all European languages except Slavic ones were included. Publications without clinical description of patients were only included in the epidemiological analysis. Forty-five reports on Caucasians with *T.b. rhodesiense* and 15 with *T.b. gambiense* infections were included in the analysis of the clinical parameters. Both species have presented with fever (*T.b. rhodesiense* 97.8% and *T.b. gambiense* 93.3%), headache (50% each) and a trypanosomal chancre (*T.b. rhodesiense* 84.4%, *T.b. gambiense* 46.7%). While sleeping disorders dominate the clinical presentation of HAT in endemic regions, there have been only rare reports in travelers: insomnia (*T.b. rhodesiense* 7.1%, *T.b. gambiense* 21.4%), diurnal somnolence (*T.b. rhodesiense* 4.8%, *T.b. gambiense* none). Surprisingly, jaundice has been seen in 24.2% of the Caucasian *T.b. rhodesiense* patients, but has never been described in HAT patients in endemic regions. These results contrast to the clinical presentation of *T.b. gambiense* and *T.b. rhodesiense* HAT in Africans in endemic regions, where the presentation of chronic *T.b. gambiense* and acute *T.b. rhodesiense* HAT is different. The analysis of 14 reports on *T.b. gambiense* HAT in Africans living in a non-endemic country shows that neurological symptoms such as somnolence (46.2%), motor deficit (64.3%) and reflex anomalies (14.3%) as well as psychiatric symptoms such as hallucinations (21.4%) or depression (21.4%) may dominate the clinical picture. Often, the diagnosis has been missed initially: some patients have even been hospitalized in psychiatric clinics. In travelers *T.b. rhodesiense* and *gambiense* present as acute illnesses and chancres are frequently seen. The diagnosis of HAT in Africans living outside the endemic region is often missed or delayed, leading to presentation with advanced stages of the disease.

## Author Summary

We systematically reviewed the existing literature, collected 95 cases of Human African Sleeping Sickness in travelers and expatriates from non-endemic countries, and observed that Sleeping Sickness in travelers generally presents as an acute febrile illness irrespective of the causative species. If present, a trypanosomal chancre or rash and itching are important diagnostic clues. Diarrhea, hepatomegaly, or jaundice are frequent and may lead to a wrong gastroenterological diagnosis. Contrary to endemic populations, where lymphadenopathy (‘*Winterbottom sign*’) or sleep disorders are hallmarks of the disease, such alterations are only occasionally found in patients from non-endemic regions. The progression of the disease to the second stage is rapid and it is only treatable with toxic drugs. The risk of a fatal outcome exists and requires a rapid diagnosis and start of treatment. The clinical presentation in immigrants with human African Trypanosomiasis (HAT) is dominated by low grade fever and neurological symptoms as well as psychiatric features. Because of the long incubation period, HAT must be considered even if the patient has left the endemic country years ago.

## Introduction

The increasing tourism to Africa is accompanied by an increasing number of imported tropical diseases including rare cases of Human African Trypanosomiasis (HAT). HAT, also known as Sleeping Sickness, is caused by the protozoan parasites *Trypanosoma brucei gambiense* (*T.b. gambiense* = West African form) and *Trypanosoma brucei rhodesiense* (*T.b. rhodesiense* = East African form), which are transmitted by the bite of the tsetse fly, *Glossina spp.*

The *T.b. gambiense* HAT is characterized by a chronic progressive course, lasting months to years, leading to death if left untreated. The *T.b. rhodesiense* HAT, however, is more acute and death occurs within weeks or months. The disease appears in two stages: the first being the early or haemo-lymphatic stage, and the second being the late or meningo-encephalitic stage, characterized by the trypanosome invasion of the central nervous system (CNS).

In endemic populations, a trypanosomal chancre (local infection at the location of the tsetse fly bite) is only seen as an exception in *T.b. gambiense*; however, it is seen in 19% of *T.b. rhodesiense* patients. Chronic and intermittent fever, headache, pruritus, lymphadenopathy, and - to a lesser extent - hepatosplenomegaly are the leading signs and symptoms of the first stage. In the second stage sleep disturbances and neuro-psychiatric disorders dominate the clinical presentation.

The diagnosis is based on the visualisation of the parasite in peripheral blood, lymph node aspirate, cerebrospinal fluid (CSF), or on polymerase chain reaction (PCR) technology, and serological tests. As treatment differs markedly between first and second stage HAT, the staging of the disease by examining the CSF is essential. According to the definition of the WHO, an elevated white blood cell count (WBC>5 cells/mm^3^) or the presence of trypanosomes in the CSF indicate a second stage disease [1].

In 1966 A.J. Duggan and M.P. Hutchinson reviewed 109 cases found in Europeans and North Americans who were infected with HAT between 1904 and 1963. They observed different clinical presentations in travelers and expatriates compared to natives of endemic regions. They reported that in Caucasians, the onset of disease is invariably acute, irrespective of the species involved. However, they documented their observations only partially with precise data.

The first objective of this study is to assess the epidemiology and the clinical presentation of HAT in travelers and expatriates from non-endemic countries and to describe the differences between the *T.b. gambiense* and *T.b. rhodesiense* cases. The second objective is to describe the clinical features of HAT patients native to endemic regions who migrated to non-endemic regions, mainly Europe and North America.

## Materials and Methods

We performed a Pubmed (MEDLINE) search of literature using the key words “Human African Trypasomiasis”, “travelers”, and “expatriates” and reviewed the available references – including the bibliographies of the retrieved references – published between 1967 and 2010 for eligible publications.

The inclusion criteria were all available publications written in European languages except Slavic languages (Dutch, English, French, German or Norwegian) on:

1. HAT patients from non-endemic regions who were diagnosed and treated in non-endemic regions
2. HAT patients from endemic regions who were diagnosed and treated in non-endemic regions

The exclusion criteria were publications in Slavic or Asian languages. Reports with insufficient clinical description were only included in the epidemiological analyses.

Additionally, unpublished HAT cases reported on ProMED-mail (URL: http://www.promedmail.org) and unpublished cases from the personal archive of the authors were included if they met the inclusion criteria.

The patients were allocated to the following groups:

1. Travelers: Patients with non-endemic background, namely travelers, expatriates, and long-time residents who became infected in Africa and treated in their country of origin
2. Immigrants: Migrated native Africans who were diagnosed and treated in non-endemic regions

African HAT patients diagnosed and treated in endemic regions were used for comparison. The species determination was based on geographical localization. We collected all available data on epidemiological background, clinical manifestations, laboratory parameters, applied diagnostic methods, and treatment regimens of the included cases. After extracting the data from the publications fulfilling the inclusion criteria, the clinical and laboratory characteristics as well as the neurological symptoms between the 3 groups (*T.b. rhodesiense* infected travelers, *T.b. gambiense* infected travelers and HAT infected immigrants) were compared using the Fisher exact test (for binary variables) and the Kruskal Wallis test (for continuous variables). A p-value<0.05 was considered statistically significant. The statistical analyses were performed using STATA 10.1 (Stata Corp LP, College Station, TX, USA).

## Results

One hundred twenty-one cases met the inclusion criteria: 99 cases from the literature search, 19 cases reported on ProMED-mail, and three cases from the personal archives of the authors. The references of all cases are listed in text S1.

**Figure 1:**
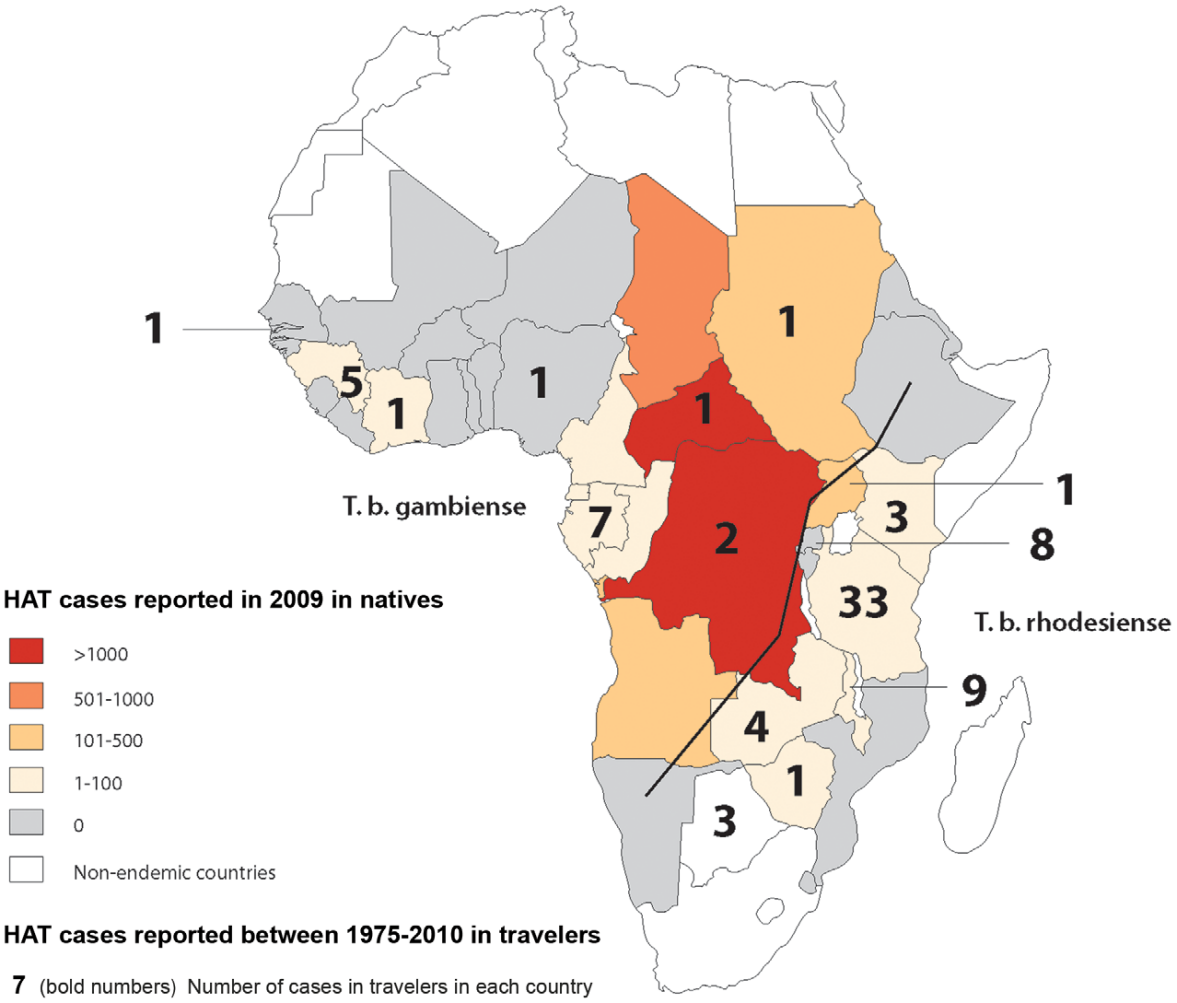
HAT in endemic and non-endemic populations (traveler). In 14 patients, more than one country was estimated to be country of infection: Kenya or Tanzania: 7; Cameroon or Congo: 2; Nigeria or Gabon: 1; Zambia or Botswana: 1, Zambia, Zimbabwe or Tanzania: 1; Namibia, Mozambique or Malawi: 1, East Africa: 1 (these patients are not counted in the figure). The black line divides the endemic regions of *T.b. gambiense* and *T.b. rhodesiense* HAT. Modified data from Simarro P. et al. 2011.

**Figure 2:**
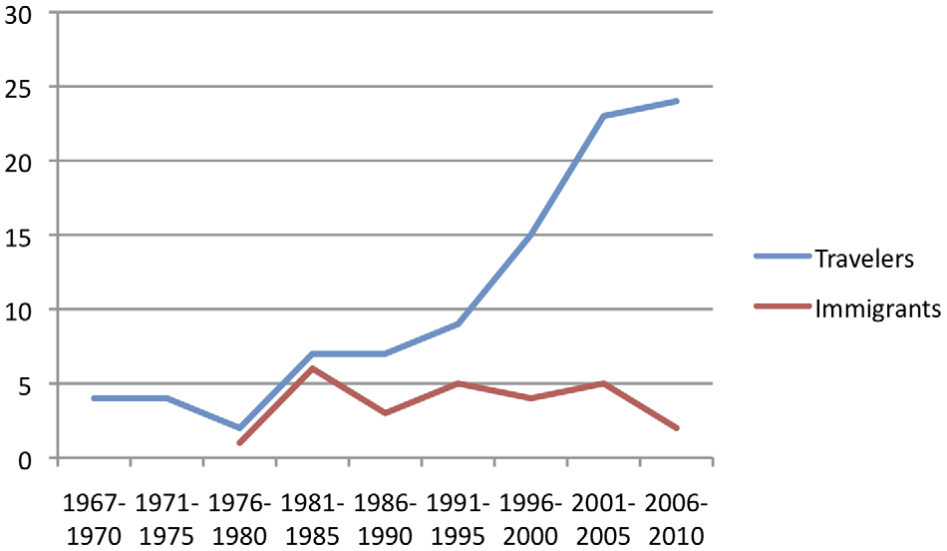
Sleeping sickness reported cases over the years in travelers. Note: The date refers to the date of diagnosis, not to the publication date of the case.

The epidemiological data are summarized in Figures 1 and 2. The description of the groups, the data on clinical findings, laboratory results, diagnostic methods and response to treatment are shown in Tables 1, 2, 3, 4, 5, 6.

**Table 1:** Description of the groups.

	Number	Travelers		Immigrants
		*T.b. rhodesiense*	*T.b. gambiense*	*T.b. gambiense*
Total	121	74	21	26
Male/female	76/36	43/22	19/2	14/12
Age (mean)	39.4	42.3	39.0	32.4
Stage 1/Stage 2	67/47	53/17	14/7	0/23

**Table 2:** Clinical signs and symptoms.

	Travelers		Immigrants	Fisher test p-value
	*T.b. rhodesiense*	*T.b. gambiense*	*T.b. gambiense*	
	n = 45	n = 15	n = 11	
	%	%	%	
Moderate fever (37.5–38.5°C)	31.1	40.0	54.6	0.336
High fever (>38.5°C)	66.7	53.3	36.4	0.150
Chills	28.9	20	0	0.108
Trypanosomal chancre	84.4*	46.7*	9.1	**0.0001** *0.0034
Trypanosomal rash	28.9	33.3	0	0.79
Headache	48.9	53.3	36.4	0.698
Pruritus	4.4	20.0	9.1	0.102
Weight loss	8.9*	40.0*	18.2	**0.020** *0.005
Diarrhea	17.8	6.7	0	0.342
Nausea /vomiting	37.8	20	0	0.029
Myalgia	22.2	20	27.3	0.921
Jaundice	24.2*	0*	0	**0.028** *0.034
Lymphadenopathy generalized	13.3*	40.0*	66.7	**0.001** *0.025
Lymphadenopathy satellite to chancre	26.7	33.3	8.3	0.316
Splenomegaly	27.3*	60.0*	27.3	0.067*0.022
Hepatomegaly	17.8	33.3	27.3	0.388
Tachycardia (>100/min)	11/20 = 55	5/8 = 62.5	No data	0.510
Hypotension (systolic<100)	3/14 = 21.4	1/5 = 20	No data	1.000

A * behind a number signifies a significant difference between *T.b. gambiense* and *T.b. rhodesiense* travelers.

**Table 3:** Neurological and psychiatric symptoms.

	Travelers		Immigrants	Fisher test p-value
	*T.b. rhodesiense*	*T.b. gambiense*	*T.b. gambiense*	
	n = 42	n = 14	n = 14	
	%	%	%	
Personality change	0	0	14.3	0.075
Hallucinations	4.8	0	21.4	0.102
Depression	0	0	21.4	**0.013**
Tremor	4.9	14.3	21.4	0.131
Abnormal reflexes	0	7.7	23.1	**0.012**
Reduced level of consciousness	2.5	0	42.9	**0.0001**
Extrapyramidal symptoms	2.5	0	14.3	0.202
Sensory deficit	0	7.7	14.3	0.064
Motor deficit	0*	15.4*	64.3	**0.001** *0.0115
Daytime somnolence	4.8	0	46.2	**0.001**
Nighttime insomnia	7.1	21.4	0	0.168
Daytime somnolence & nighttime insomnia	2.6	7.1	23.1	**0.034**

**Table 4:** Laboratory parameters.

	Travelers		Immigrants	Fisher test p-value
	*T.b. rhodesiense*	*T.b. gambiense*	*T.b. gambiense*	
Elevated inflammatory parameter§	8/8 = 100%	8/9 = 88.9%	6/6 = 100%	1.000
WBC<3.54×10^3^/mm^3^	15/43 = 34.9%	3/7 = 42.9%	1/12 = 8.3%	0.164
WBC>9.06×10^3^/mm^3^	5/43 = 11.6%	2/7 = 28.6%	3/12 = 25%	0.289
Hb<12 g/dl (female) & <13.3 g/dl (male)	18/34 = 52.9%	8/9 = 88.9%	11/11 = 100%	**0.003** *0.0489
Platelets<165 000/mm^3^	37/42 = 88.1%	3/5 = 60%	5/8 = 62.5%	0.082
Elevated liver enzymes#	31/35 s = 80.7%	1/3 = 33.3%	0/3 = 0%	**0.005**
Total bilirubin>1.3 mg/dl	17/22 = 77.3%	No data	0/2 = 0%	0.076
Creatinin>0.9 mg/dl (female) & >1.2 (male)	24/29 = 82.8%	0/3 = 0%	0/4 = 0%	**0.0016** *0.001

Normal reference of value out of Harrison's Online (http://www.accessmedicine.com/popup.aspx?aID=2904606, date: 10.11.2010).

**§:** At least one of the following parameters was elevated: C-reactive protein (CRP)>3.0 mg/L; erythrocyte sedimentation rate female>20 mm/h, male>15 mm/h.

# At least one of the following parameters was elevated: Alanin aminotransferase (SGOT, ALAT)>7–41 U/l; Aspartate aminotransferase (SGPT, ASAT)>12–38 U/l; Alkaline Phosphatase (ALP)>60–170 U7l).

**Table 5:** Diagnostic methods.

	Number	Travelers		Immigrants
		*T.b. rhodesiense*	*T.b. gambiense*	*T.b. gambiense*
	*(n = 121)*	*(n = 74)*	*(n = 21)*	*(n = 26)*
Blood smear	76	64	9	3
Buffy coat test	1	0	1	0
PCR of the buffy coat	1	0	0	1
Chancre fluid aspirate	2	2	0	0
Lymph node aspirate	1	0	1	0
Bone marrow aspirate	1	0	1	0
CSF microscopy	12	0	2	10
Serology and cytology /histology (mott cells)	3	0	0	3
No data	24	8	7	9

**Table 6:** Response to treatment.

	Number	Travelers		Immigrants
		*T.b. rhodesiense*	*T.b. gambiense*	*T.b. gambiense*
	*(n = 121)*	*(n = 74)*	*(n = 21)*	*(n = 26)*
Cure	95	58	19	18
Death	5	3	0	2
Relapse	7	3	2	2
No data	14	10	0	4

Forty-five reports on travelers infected with *T.b. rhodesiense* and 15 reports on travelers infected with *T.b. gambiense* were included in the analysis of the clinical parameters. Both species presented with fever (*T.b. rhodesiense* 98%; *T.b. gambiense* 93%), headache (close to 50% each), and a trypanosomal chancre (*T.b. rhodesiense* 84%; *T.b. gambiense* 47%). While insomnia and diurnal somnolence dominate the clinical presentation of HAT in endemic regions, insomnia (*T.b. rhodesiense* 7%, *T.b. gambiense* 21%) or diurnal somnolence (*T.b. rhodesiense* 5%, *T.b. gambiense* none) have only rarely been described in travelers. Surprisingly, jaundice has been reported in 24% of *T.b. rhodesiense* infected travelers. The analysis of 14 reports on HAT infected immigrants (all infected with *T.b. gambiense*) shows that neurological symptoms such as somnolence (46%), motor deficit (64%) and reflex anomalies (14.3%) as well as psychiatric symptoms such as hallucinations (21%) or depression (21%) may dominate the clinical picture. Often, the diagnosis has been missed initially: some patients have even been hospitalized in psychiatric clinics.

While 83% (34/41) of *T.b. rhodesiense* infected travelers had a history of staying <30 days and 17% (7/41)>30 days within endemic regions, 29% (4/14) of *T.b. gambiense* infected travelers had spent <30 days and 71% (10/14)>30 days within endemic regions.

The activities determining the risk of exposure in travelers infected with *T.b. rhodesiense* included: visits to game parks 44/57 (77.2%), hunting safaris 5/57 (8.8%), military missions 4/57 (5.2%), business trips 2/57 (3.5%), and being expatriate 2/57 (3.5%). The main activities determining the risk of exposure in travelers infected with *T.b. gambiense* were: traveling as tourists 3/16 (18.8%), business trips 5/16 (31.3%) and being expatriate 8/16 (50%).

The putative incubation period was estimated to be ≤14 days in 39/54 (72%) and 15 to 21 days in 11/54 (20%) travelers infected with *T.b. rhodesiense*. In 4/54 (7%) patients, the time of infection was not precisely known, but - considering the maximal range of exposure - less than 30 days. The incubation period in travelers infected with *T.b. gambiense* has been <21 days in 4/7 (57%), 21 to 30 days in 1/7 (14%) and >3 months in 2/7 (29%) patients. The incubation period in immigrants was estimated - considering the time of leaving the endemic region and the appearance of the first symptom - to be <5 months in 3/10 (30%), >two years in 6/10 (60%) and even >7 years in 1/10 (10%) patients.

ECG findings have not been routinely described among the reviewed cases. In 14 travelers the following abnormalities have been reported: in the *T.b. rhodesiense* group 4/10 (40%) have been normal, 1/10 (10%) has shown sinustachycardia, 4/10 (40%) ST- and T- wave abnormalities, and 1/10 (10%) a first degree atrioventricular block. In the *T.b. gambiense* group, 1/4 (25%) has been normal, 2/4 (50%) have presented a first degree atrioventricular block, and 1/4 (25%) a third degree atrioventricular block.

One single investigation of peripheral blood smears has established the diagnosis in 57/64 (89%) *T.b. rhodesiense* infected travelers and in 5/9 (56%) *T.b. gambiense* infected travelers. Repeated examinations have been necessary in eleven patients. In four immigrants, trypanosomes could not be detected by microscopical analysis of peripheral blood or CSF. In one patient, the diagnosis has been established by positive serology and detection of “Mott” cells in bone marrow aspirates; in a second patient by finding “Mott” cells in a brain biopsy; in a third patient by positive serology and histological findings consistent with *T.b. gambiense* infection; and in a fourth patient by using a PCR assay of the buffy coat and CSF samples.

Travelers infected with *T.b. rhodesiense* in the first stage have been treated with suramin (42/53 patients (79,2%)). Because suramin was not available, the following alternatives have been used to treat 11 patients: pentamidine (5/53 patients (9.4%)), pentamidine followed by suramin (5/53 patients (9.4%)), and melarsoprol (1/53 patient (1.9%)). All 17 second stage *T.b. rhodesiense* patients have been treated with melarsoprol. Out of all *T.b. rhodesiense* infected travelers, three patients died and three had relapses. Two have died of an encephalopathic syndrome due to melarsoprol and one due to severe complications (disseminated intravascular coagulation, cardiac arrhythmia, pneumonia, and generalized seizure). Among the *T.b. gambiense* infected travelers, no patient has died.

*T.b. gambiense* infected patients (both travelers and immigrants) with first stage HAT have been treated with pentamidine (10/14 patients (71.4%)), suramin (2/14 patients (14.3%)) and eflornithin (2/14 patients (14.3%)). The patients in second stage HAT have been treated with eflornithine (17/29 patients (58.6%)) or melarsoprol (12/29 patients (41.4%)).

## Discussion

In contrast to African patients in whom the clinical presentation of chronic *T.b. gambiense* and acute *T.b. rhodesiense* infections are distinctly different, both species present as acute febrile illness in travelers with few differences. Sleeping disorders and neurological findings do not dominate the clinical presentation.

### Fever

Patients infected with both species have presented with fever ≥37.5°C (*T.b. rhodesiense* 98%; *T.b. gambiense* 93%). The fever has been >38.5°C in more than half of the patients irrespective of *species*: *T.b. rhodesiense* (67%), *T.b. gambiense* (53%). In endemic patients high fever has less frequently been reported in *T.b. gambiense* [2]–[5] than in *T.b. rhodesiense* HAT [6]–[8]. Possible explanations for this difference may be due to genetic factors or a lack of previous exposure to non-human-pathogenic forms of trypanosomes, possibly contributing to the development of partial immunity. Sporadic cases of such human infections with putative non-human-pathogenic trypanosomes have been reported [9]–[11].

### Sleeping disorders and other neurological disorders

A key finding of this review is that the classical sleep disorders of HAT and neurological findings are not a hallmark in travelers, irrespective of species. Sleep disorders have only been present in a minority of *T.b. rhodesiense* infected travelers. Nighttime insomnia has only been observed in 21% of *T.b. gambiense* infected travelers. Apart from tremor and motor deficits in 15% of *T.b. gambiense* infected patients neurological and psychiatric findings have not been reported in travelers. In general, the eponymous sleep disorders of second stage HAT are mostly seen in patients infected with *T.b. gambiense* (see Table 7) [2]. This is commonly explained by the prolonged course of second stage disease [2]. Further, the incidence of neurological disorders increases with the evolution of the disease in both species [2], [3]. Since most of the travelers have been in the first stage and had a short duration of the disease, sleep disorders and neuropsychiatric findings may not have been developed at the time of the first clinical assessment. In addition, nighttime insomnia may have been overlooked and underreported because of its relatively benign character in an otherwise acute and often life threatening disease. Somnolence is a common and unspecific symptom in any severe febrile disease and might therefore be underreported. The absence or presence of sleep disorders or other neuropsychiatric findings in travelers may not be decisive for the assessment of HAT. In contrast, somnolence and the classical disruption of the sleep cycle with daytime somnolence and nighttime insomnia, as well as other neuropsychiatric findings, are frequently observed in immigrants.

**Table 7:** Signs and symptoms according to stage and affected population.

	*Population*	*T.b. gambiense*		*T.b. rhodesiense*	
		First stage	Second stage	First stage	Second stage
**Incubation period**	Natives	18 months [34]	18 months [34]	1–3 weeks	few weeks
	Travelers	75% <1 month	No data	<3 weeks	>4 weeks
**Chancre**	Natives	<5% [12], [13], [35]	0 [8], [11]	5–26% [3], [5], [14], [15], [36]	0 [3], [16]
	Travelers	55.6%	33%	87.9%	75%
**Trypanosomal rash**	Natives	0% [12], [13], [35]	0 [8], [11]	0% [5], [14], [36]	0 [3], [16]
	Travelers	22.2%	50%	24.4%	41.7%
**Fever (≥37.5°C)**	Natives	10–20% [12], [13], [35]	10–40% [8], [11]–[13], [19], [35], [37], [38]	28–90% [3], [5]	18–37% [3], [15], [16], [36] −72% [14]
	Travelers	88.9%; >38.5°: 55.6%	100%; >38.5°: 50%	100%; >38.5°: 72.7%	91.7%; >38.5°: 50%
**Lymphadenopathy**	Natives	79–95% [12], [13], [35]	56–85% [8], [11]–[13], [35]	21% [3]	51–80% [3], [14]–[16], [36]
	Travelers	Generalized 33.3%Satellite (to chancre) 22.2%	Generalized 50%Satellite (to chancre) 50%	Generalized 6.1%Satellite (to chancre) 30.3%	Generalized 33.3%Satellite (to chancre) 16.7%
**Sleeping disorder**	Natives	Somnolence 18%Insomnia 73% [13]	Somnolence 29–41% [8],[11]Insomnia 25–57% [8], [11], [13]	Somnolence 25–33% [3], [5]	Somnolence 54–66%Insomnia 28–64% [3], [14]–[16], [36]
	Travelers	Somnolence 0%Insomnia 28.6%	Somnolence 0%Insomnia 16.7%	Somnolence 0%Insomnia 6.7%	Somnolence 16.7%Insomnia 8.3%
**Pruritus**	Natives	29–33% [12], [13], [39]	17–57% [8], [11]–[13], [39]	0% [3]	6–53% [14]–[16]
	Travelers	22.2%	16.7%	3%	8.3%
**Headache**	Natives	51–80% [12], [39]	38–79% [8], [11]–[13], [39], [40]	96% [5]	51–80% [3], [16]
	Travelers	55.5%	50%	42.4%	66.7%
**Hepatomegaly**	Natives	0–20% [12], [13], [39]	7–17% [8], [13]	0–40% [3]	6–30% [3], [16]
	Travelers	22.2%	50%	15.6%	25%
**Splenomegaly**	Natives	9–27% [12], [13], [39]	5–19% [8], [12], [13]	0–36% [3]	16–58% [3], [16]
	Travelers	55.6%	66.7%	30.3%	8.3%
**Tremor**	Natives	5% [35]	19–21% [8], [41]	17–61% [3]	16–67% [3], [16]
	Travelers	14.3%	0%	0%	16.7%
**Neurological disorder**	Natives	<20% [12], [13]	20–40% [11], [13]	<20% [3]	50–58% [3], [16]
	Travelers	25%	33.3%	0%	8.3%
**Psychiatric disorders**	Natives	<10%	25% [11]	17% [36]	15–22% [3], [16]
	Travelers	0%	0%	3.3%	8.3%
**Kidney impairment**	Natives	rare [42]	rare [17], [42]	unknown [3]	unknown
	Travelers	0%	0%	85%	77.7%

### Chancre and other skin alterations

While in endemic populations a trypanosomal chancre is rarely seen [4], [5], a trypanosomal chancre is a key finding in 84% of *T.b. rhodesiense* and 47% of *T.b. gambiense* infected travelers (see table 7). The presence of a trypanosomal rash might be an important diagnostic clue; this exanthema, which may appear at any time after the first febrile episode, consists of blotchy irregular erythematous macules with a diameter of up to 10 cm. A large proportion of the macules develop a central area of normal-colored skin, giving the rash a circinate or serpiginous outline. The trunk is mainly affected and the erythema is seldom pronounced. The rash is evanescent, fading in one place and reappearing in another over a period of several weeks. It is not tender and does not itch [6], [7]. In our study, such a trypanosomal rash has been present in approximately one third of the travelers, irrespective of the species.

Pruritus is a well known symptom of Western [2], [8], [12], [13] and – to a lesser extent [14]–[16] - Eastern HAT. Among our reviewed cases, pruritus has been present in 20% of the travelers infected with *T.b. gambiense*, but only in 4% of the travelers infected with *T.b. rhodesiense*. In the differential diagnosis of fever in returning travelers pruritus and the respective scratch marks might constitute valuable diagnostic clues for *T.b. gambiense* HAT.

### Gastrointestinal findings

Liver involvement with clinical hepatomegaly and elevated liver function tests (LFT) is a known feature of HAT [2], [3], [5], [8], [15]–[18]. Hepatomegaly has frequently been reported among our reviewed immigrants and travelers. Interestingly 24% of the travelers infected with *T.b. rhodesiense* have been with jaundice, a sign that has only occasionally been reported in HAT infected immigrants [2], [3], [5], [8], [15]–[19] (table 2). Our review highlights the fact that *T.b. rhodesiense* HAT should be included in the differential diagnoses of febrile travelers presenting with jaundice or abnormal LFT.

### Further clinical findings

No textbook on the clinical description of the Human African Sleeping Sickness will omit the classical description of the “Winterbottom's sign”, the cervical lymphadenopathy, which is mostly described as a characteristic trait of HAT. Among the reviewed HAT cases, general lymphadenopathy has been reported in a majority of *T.b. gambiense* infected immigrants (Table 2). In travelers, however, lymphadenopathy is absent in the majority of cases and does therefore not facilitate diagnosis.

Cardiac involvement with myopericarditis, arrythmias and ECG changes (QT_c_ prolongation, repolarisation changes, and low voltage) has been observed in endemic HAT patients [20]. While clinically relevant heart failure is rarely observed in *T.b. gambiense* HAT [21], [22], myopericarditis appears to play an important role in the clinical course and fatal outcome of *T.b. rhodesiense* infected endemic population [22]. The few data on cardiac involvement in travelers include myopericarditis [23], [24], transient second degree [25] and third degree atrioventricular block [26], and ventricular premature captures (class Lown IV b) [26].

The kidney function has been impaired in most *T.b. rhodesiense* travelers (83%). In contrast to endemic populations, where endocrine disorders of the thyroid and adrenocortical function [17], [27], [28] are described, no such alterations have been reported in travelers. The white blood cell count has been mostly normal or even low, and most patients presented thrombocytopenia.

### Epidemiology

Numbers of HAT cases have markedly decreased in endemic countries in the past decade – after an increase between 1969 and 2000 [29] –, while the number of reported HAT cases in non-endemic countries shows a considerable increase (Figure 2). When interpreting these epidemiological developments, it is important to consider the essential differences between *T.b. gambiense* and *T.b. rhodesiense* HAT. In endemic regions *T.b. gambiense* is responsible for more than 95% of all HAT patients [30]; in travelers only a minority of HAT (22%) are due to *T.b. gambiense*. Humans are considered to be the main reservoir of *T.b. gambiense* [31] and animals (e.g. pigs, dogs, etc.) play a minor role [31]. The decline of *T.b. gambiense* HAT was mainly achieved by ambitious campaigns enforcing large scale screening and treatment programs in endemic regions, targeting the human main reservoir [29]. In contrast, *T.b. rhodesiense* is primarily a zoonotic disease with wild game animals and cattle as main reservoir [31]. The increasing number of HAT observed in travelers over the last decades may be explained by the growing number of tourists who visit *T.b. rhodesiense* endemic game parks in Eastern Africa on safari and hunting trips and – to a lesser extent – by the re-emergence of HAT in the Serengeti National Park (Tanzania) in the years 2000–2005 [32].

### Incubation period

The incubation period of *T.b. gambiense* in endemic regions is difficult to assess, as the time of infection is unknown. Therefore, the estimation of the incubation period is based on patients who have left endemic countries. Since one immigrant patient developed HAT seven years after migration to a non-endemic area, the incubation period may be seven years or even longer. *T.b. gambiense* HAT should be considered in any patient from an endemic region who presents with diffuse neurological symptoms, even if already having lived abroad for a prolonged period of time.

The incubation period has been <14 days in 72% of the *T.b. rhodesiense* infected travelers, and <one month in all of them. In contrast, the incubation period in 28% of *T.b. gambiense* infected travelers has been longer, even exceeding three months.

### Diagnosis

Travelers have mostly been diagnosed by finding trypanosomes in thin or thick blood smears. Mostly, the laboratory diagnosis has easily been established in *T.b. rhodesiense* patients, usually due to suspicionfrom clinical findings. However, in *T.b. gambiense* infected patients repeated blood examinations and concentration methods have often been necessary [33]. Microscopic analysis of chancre fluid aspiration has allowed an early diagnosis in two patients. In immigrants the diagnosis has been especially difficult. Parasites have rarely been present in blood smears and analyzes of the CSF. Serologic testing or further diagnostics have been necessary.

### Treatments

The treatment of travelers follows the treatment guidelines for sleeping sickness in the endemic regions. The number of our reviewed HAT cases in travelers is too small to conclude on the toxicity or cure rates of the different drugs and treatment regimens. However, it is remarkable that five out of 17 (29.4%) patients with second stage *T.b. rhodesiense* HAT have developed an encephalopathic syndrome during treatment with melarsoprol and two of them have died. A publication bias or a delayed diagnosis and treatment could be an explanation. In some of the reviews from first stage *T.b. rhodesiense* HAT cases, suramin has initially not been available. In these cases, the treatment has been initiated using the more easily available drug pentamidine, switching to suramin later. This approach has shown good clinical response.

### Study limitations

Limitations of this study are due to the incomplete epidemiological, clinical, and laboratory data due to the retrospective nature of this study. A publication bias cannot be ruled out; however, since clinicians are impressed by this rare disease, it is likely that many cases are published, as demonstrated by the long list of references. Our search may have missed publications of travelers that had not “travel” or “traveler” as key words. First and second stage patients were not described separately because of the small number of second stage patients. Our aim was to describe the typical clinical features of HAT in travelers that might confront the physician on an initial medical consultation.

### Conclusions and recommendations

With rising number of tourists traveling to HAT endemic regions, Sleeping Sickness must be included in the differential diagnosis of any febrile patient, especially in the presence of suspicious skin manifestations or gastrointestinal manifestations.

In contrast to HAT patients in endemic regions, Sleeping Sickness in travelers generally presents as an acute febrile illness, irrespective of the causative species (see Table 7). If present, a trypanosomal chancre or rash and itching are important diagnostic clues. Diarrhea, hepatomegaly, or icterus are frequent and may lead to a wrong gastroenterologic diagnosis. In contrary to endemic populations, where lymphadenopathy (Winterbottom sign) or sleep disorders are hallmarks of the disease, such alterations are only occasionally found in travelers. The rapid progression of the disease to the second stage - in which it is only treatable with toxic drugs and the risk of a fatal outcome exists - requires a rapid diagnosis and start of treatment. The clinical presentation of HAT in immigrants is similar to the presentation of HAT patients in endemic regions (see Table 7) and is dominated by low grade fever as well as neurological and psychiatric features. Because of the long incubation period, HAT has to be considered even if the patient has left endemic regions years ago.

## Supporting Information

Checklist S1 **PRISMA checklist.**
(DOC)

Checklist S2 **PRISMA flowchart.**
(DOC)

Text S1 **References of the reviewed HAT cases and case series.**
(DOC)
