# Supplementary material for: Sleeping Sickness in Travelers - Do They Really Sleep?
Source: PLoS Negl Trop Dis. 2011 Nov 1;5(11):e1358. doi: 10.1371/journal.pntd.0001358 (PMC3206012; doi:10.1371/journal.pntd.0001358)
Supplement: Checklist S2 — PRISMA flowchart. (DOC) [file pntd.0001358.s002.doc]

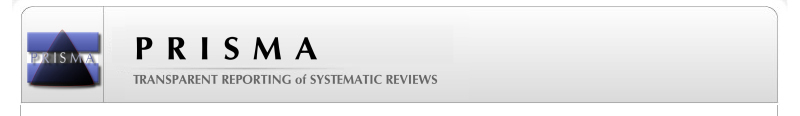
**PRISMA 2009 Flow Diagram**

**Screening**

**Included**

**Eligibility**

**Identification**

118 Records identified through database searching
(n = 118)

)

3 Additional records identified through other sources
(n = 3)

121 Records after duplicates removed
(n = 121)

121 Records screened
(n = 121)

0 Records excluded
(n = 0)

121 Full-text articles assessed for eligibility
(n = 121)

0 Full-text articles excluded, with reasons
(n = 0)

121 Studies included in qualitative synthesis
(n = 121)

121 Studies included in quantitative synthesis (meta-analysis)
(n = 121)
