# Supplementary material for: Sleeping Sickness in Travelers - Do They Really Sleep?
Source: PLoS Negl Trop Dis. 2011 Nov 1;5(11):e1358. doi: 10.1371/journal.pntd.0001358 (PMC3206012; doi:10.1371/journal.pntd.0001358)
Supplement: Text S1 — References of the reviewed HAT cases and case series. (DOC) [file pntd.0001358.s003.doc]

**References of reviewed HAT cases (cases and case series)**

1. Bakken JS, Arroe M (1985) [African trypanosomiasis. Report on a patient]. Tidsskr Nor Laegeforen 105: 1501-1502.

2. Bedat-Millet AL, Charpentier S, Monge-Strauss MF, Woimant F (2000) [Psychiatric presentation of human African trypanosomiasis: overview of diagnostic pitfalls, interest of difluoromethylornithine treatment and contribution of magnetic resonance imaging]. Rev Neurol (Paris) 156: 505-509.

3. Benhamou PH, Chandenier J, Schechter PJ, Epelbaum S, Tell GP, Haegele KD, Pautard JC, Piussan C (1989) [African trypanosomiasis in children treated with eflornithine. A case]. Presse Med 18: 1199-1202.

4. Bisoffi Z, Beltrame A, Monteiro G, Arzese A, Marocco S, Rorato G, Anselmi M, Viale P (2005) African trypanosomiasis gambiense, Italy. Emerg Infect Dis 11: 1745-1747.

5. Blanchot I, Dabadie A, Tell G, Guiguen C, Faugere B, Plat-Pelle AM, Roussey M (1992) [Recurrent fever episodes in an African child: diagnostic difficulties of trypanosomiasis in France]. Pediatrie 47: 179-183.

6. Blumberg L (2005) ProMed-mail 20051224.3675.

7. Blumberg L (2007) ProMed-mail 20070212.0532.

8. Blumberg L (2007) ProMed-mail 20071120.3754.

9. Boller K (1977) [African sleeping sickness in Switzerland (trypanosomiasis rhodesiensis)]. Schweiz Med Wochenschr 107: 1706-1708.

10. Bourgeade A, Nosny Y, Faugere B, Pene P (1985) [African trypanosomiasis of icterohemorrhagic form]. Bull Soc Pathol Exot Filiales 78: 908-913.

11. Braakman HM, van de Molengraft FJ, Hubert WW, Boerman DH (2006) Lethal African trypanosomiasis in a traveler: MRI and neuropathology. Neurology 66: 1094-1096.

12. Braendli B, Dankwa E, Junghanss T (1990) [East African sleeping sickness (Trypanosoma rhodesiense infection) in 2 Swiss travelers to the tropics]. Schweiz Med Wochenschr 120: 1348-1352.

13. Buyse D, Van den EJ, Vervoort T, Van den EE (1996) [Sleeping sickness as an import pathology following a stay in Zaire]. Acta Clin Belg 51: 409-411.

14. Callens S, van Wijngaerden E, Clerinx J, Colebunders R (2003) [Three patients with African sleeping sickness following a visit to Tanzania]. Ned Tijdschr Geneeskd 147: 581.

15. Checkley AM, Pepin J, Gibson WC, Taylor MN, Jager HR, Mabey DC (2007) Human African trypanosomiasis: diagnosis, relapse and survival after severe melarsoprol-induced encephalopathy. Trans R Soc Trop Med Hyg 101: 523-526.

16. Claessen FA, Blaauw GJ, van der Vorst MJ, Ang CW, van Agtmael MA (2010) Tryps after adventurous trips. Neth J Med 68: 144-145.

17. Clernix J. (2010) Personal communication.

18. Cochran R, Rosen T (1983) African trypanosomiasis in the United States. Arch Dermatol 119: 670-674.

19. Croft AM, Jackson CJ, Friend HM, Minton EJ (2006) African trypanosomiasis in a British soldier. J R Army Med Corps 152: 156-160.

20. Damian MS, Dorndorf W, Burkardt H, Singer I, Leinweber B, Schachenmayr W (1994) [Polyneuritis and myositis in Trypanosoma gambiense infection]. Dtsch Med Wochenschr 119: 1690-1693.

21. Darby JD, Huber MG, Sieling WL, Spelman DW (2008) African trypanosomiasis in two short-term Australian travelers to Malawi. J Travel Med 15: 375-377.

22. Dupont B, Charmot G, Lapresle C (1979) [Trypanosomiasis presenting with trypanids and complicated by myopericarditis (author's transl)]. Nouv Presse Med 8: 1579-1581.

23. Ezzedine K, Darie H, Le Bras M, Malvy D (2007) Skin features accompanying imported human African trypanosomiasis: hemolymphatic Trypanosoma gambiense infection among two French expatriates with dermatologic manifestations. J Travel Med 14: 192-196.

24. Faust SN, Woodrow CJ, Patel S, Snape M, Chiodini PL, Tudor-Williams G, Hermione Lyall EG (2004) Sleeping sickness in brothers in london. Pediatr Infect Dis J 23: 879-881.

25. Freedman D (2000) ProMed-mail 20001106.1935.

26. Freedman D (2010) ProMed-mail 20100915.3338.

27. Gill DS, Chatha DS, Carpio-O'Donovan R (2003) MR imaging findings in African trypansomiasis. AJNR Am J Neuroradiol 24: 1383-1385.

28. Ginsberg R, Ackley A, Stoner E, Lee L (1986) African sleeping sickness presenting in an American emergency department. Ann Emerg Med 15: 86-88.

29. Gopalakrishnan R, Easow JM (2003) East African sleeping sickness in Chennai. J Assoc Physicians India 51: 302-303.

30. Grau Junyent JM, Rozman M, Corachan M, Estruch R, Urbano-Marquez A (1987) An unusual course of west African trypanosomiasis in a Caucasian man. Trans R Soc Trop Med Hyg 81: 931-932.

31. Hart W, Slee PH, Schipper HG, Koopmans RP, Kager PA (2004) [Clinical reasoning and decision making in practice. A depressive foreign woman with symptoms of malaise]. Ned Tijdschr Geneeskd 148: 771-776.

32. Hope-Rapp E, Moussa CO, Klement E, Danis M, Bricaire F, Caumes E (2009) [Double trypanosomal chancre revealing West African trypanosomiasis in a Frenchman living in Gabon]. Ann Dermatol Venereol 136: 341-345.

33. Iborra C, Danis M, Bricaire F, Caumes E (1999) A traveler returning from Central Africa with fever and a skin lesion. Clin Infect Dis 28: 679-680.

34. Jelinek T (2001) ProMed-mail 20010316.0535.

35. Jelinek T (2009) ProMed-mail 20090724.2613.

36. Jelinek T, Bisoffi Z, Bonazzi L, van Thiel P, Bronner U, de Frey A, Gundersen SG, McWhinney P, Ripamonti D (2002) Cluster of African trypanosomiasis in travelers to Tanzanian national parks. Emerg Infect Dis 8: 634-635.

37. Jelinek T (2001) ProMed-mail 20011016.2542.

38. Kager PA, Schipper HG, Stam J, Majoie CB (2009) Magnetic resonance imaging findings in human African trypanosomiasis: a four-year follow-up study in a patient and review of the literature. Am J Trop Med Hyg 80: 947-952.

39. Kirchhoff LV (1998) Use of a PCR assay for diagnosing African trypanosomiasis of the CNS: a case report. Cent Afr J Med 44: 134-136.

40. Kirrstetter M, Lerin-Lozano C, Heintz H, Manegold C, Gross WL, Lamprecht P (2004) [Trypanosomiasis in a woman from Cameroon mimicking systemic lupus erythematosus]. Dtsch Med Wochenschr 129: 1315-1317.

41. Klaassen B (2008) ProMed-mail 20081103.3455.

42. Kumar N, Orenstein R, Uslan DZ, Berbari EF, Klein CJ, Windebank AJ (2006) Melarsoprol-associated multifocal inflammatory CNS illness in African trypanosomiasis. Neurology 66: 1120-1121.

43. Landron C, Roblot F, Le Moal G, Becq-Giraudon B (2003) African trypanosomiasis acquired in an urban area. Eur J Intern Med 14: 390-391.

44. Legros F (2006) [Trypanosomiase humaine africaine : recensement des cas d'importation observés en France, 1980-2004]. Bulletin épidémiologique hebdomandaire (BEH) 7: 57-59.

45. Liu AP, Chou S, Gomes L, Ng T, Salisbury EL, Walker GL, Packham DR (2010) Progressive meningoencephalitis in a Sudanese immigrant. Med J Aust 192: 413-416.

46. Loscher T, Nothdurft HD, Taelman H, Boogaerts M, Omar M, von Sonnenburg F (1989) [Sleeping sickness in German travelers to the tropics]. Dtsch Med Wochenschr 114: 1203-1206.

47. Maddocks S, O'Brien R (2000) Images in clinical medicine. African trypanosomiasis in Australia. N Engl J Med 342: 1254.

48. Malesker MA, Boken D, Ruma TA, Vuchetich PJ, Murphy PJ, Smith PW (1999) Rhodesian trypanosomiasis in a splenectomized patient. Am J Trop Med Hyg 61: 428-430.

49. Malgorzata P (2009) ProMed-mail 20090810.2844.

50. Malvy D, Djossou F, Weill FX, Chapuis P, Longy-Boursier M, Le Bras M (2000) Guess what! Human West African trypanosomiasis with chancre presentation. Eur J Dermatol 10: 561-562.

51. McGovern TW, Williams W, Fitzpatrick JE, Cetron MS, Hepburn BC, Gentry RH (1995) Cutaneous manifestations of African trypanosomiasis. Arch Dermatol 131: 1178-1182.

52. Mendonca MM, Rasica M, van Thiel PP, Richter C, Kager PA, Wismans PJ (2002) [Three patients with African sleeping sickness following a visit to Tanzania]. Ned Tijdschr Geneeskd 146: 2552-2556.

53. Montmayeur A, Brosset C, Imbert P, Buguet A (1994) [The sleep-wake cycle during Trypanosoma brucei rhodesiense human African trypanosomiasis in 2 French parachutists]. Bull Soc Pathol Exot 87: 368-371.

54. Moore AC, Ryan ET, Waldron MA (2002) Case records of the Massachusetts General Hospital. Weekly clinicopathological exercises. Case 20-2002. A 37-year-old man with fever, hepatosplenomegaly, and a cutaneous foot lesion after a trip to Africa. N Engl J Med 346: 2069-2076.

55. Moore DA, Edwards M, Escombe R, Agranoff D, Bailey JW, Squire SB, Chiodini PL (2002) African trypanosomiasis in travelers returning to the United Kingdom. Emerg Infect Dis 8: 74-76.

56. Myrvang B, von der LB (2002) [African trypanosomiasis--a rare imported disease]. Tidsskr Nor Laegeforen 122: 33-34.

57. Nadjm B, Van Tulleken C, Macdonald D, Chiodini PL (2009) East African trypanosomiasis in a pregnant traveler. Emerg Infect Dis 15: 1866-1867.

58. Niederau C, Gunther B, Erkenbrecht J, Strohmeyer G (1994) [Fever after travel to the tropics in Africa: the etiology is not always malaria]. Internist (Berl) 35: 755-758.

59. Nutman T (2005) ProMed-mail 20050713.1989.

60. Oscherwitz SL (2003) East African trypanosomiasis. J Travel Med 10: 141-143.

61. Otte JA, Nouwen JL, Wismans PJ, Beukers R, Vroon HJ, Stuiver PC (1995) [African sleeping sickness in The Netherlands]. Ned Tijdschr Geneeskd 139: 2100-2104.

62. Panosian CB, Cohen L, Bruckner D, Berlin G, Hardy WD (1991) Fever, leukopenia, and a cutaneous lesion in a man who had recently traveled in Africa. Rev Infect Dis 13: 1131-1138.

63. Petru AM, Azimi PH, Cummins SK, Sjoerdsma A (1988) African sleeping sickness in the United States. Successful treatment with eflornithine. Am J Dis Child 142: 224-228.

64. Ponce-de-Leon S, Lisker-Melman M, Kato-Maeda M, Gamboa-Dominguez A, Ontiveros C, Behrens RH, Gonzalez-Ruiz A (1996) Trypanosoma brucei rhodesiense infection imported to Mexico from a tourist resort in Kenya. Clin Infect Dis 23: 847-848.

65. Quinn TC, Hill CD (1983) African trypanosomiasis in an American hunter in East Africa. Arch Intern Med 143: 1021-1023.

66. Raffenot D, Rogeaux O, Goer BD, Doche C, Tous J (2000) [Infectious mononucleosis or sleeping sickness?]. Ann Biol Clin (Paris) 58: 94-96.

67. Richter J. (2010) Personal communication.

68. Ripamonti D, Massari M, Arici C, Gabbi E, Farina C, Brini M, Capatti C, Suter F (2002) African sleeping sickness in tourists returning from Tanzania: the first 2 Italian cases from a small outbreak among European travelers. Clin Infect Dis 34: E18-E22.

69. Sabbah P, Brosset C, Imbert P, Bonardel G, Jeandel P, Briant JF (1997) Human African trypanosomiasis: MRI. Neuroradiology 39: 708-710.

70. Sahlas DJ, MacLean JD, Janevski J, Detsky AS (2002) Clinical problem-solving. Out of Africa. N Engl J Med 347: 749-753.

71. Sanner BM, Doberauer C, Tepel M, Zidek W (2000) Fulminant disease simulating bacterial sepsis with disseminated intravascular coagulation after a trip to East Africa. Intensive Care Med 26: 646-647.

72. Scott JA, Davidson RN, Moody AH, Bryceson AD (1991) Diagnosing multiple parasitic infections: trypanosomiasis, loiasis and schistosomiasis in a single case. Scand J Infect Dis 23: 777-780.

73. Sinha A, Grace C, Alston WK, Westenfeld F, Maguire JH (1999) African trypanosomiasis in two travelers from the United States. Clin Infect Dis 29: 840-844.

74. Spencer HC, Jr., Gibson JJ, Jr., Brodsky RE, Schultz MG (1975) Imported African trypanosomiasis in the United States. Ann Intern Med 82: 633-638.

75. Stephan C, Just-Nuebling G, Fichtlscherer S, Kriener S, Brodt HR (2002) Winterbottom's sign and hypertrophic cardiomyopathy. Scand J Infect Dis 34: 544-545.

76. Sterner G, Nasander L (1977) African trypanosomiasis: a danger for tourists visiting Gambia? Scand J Infect Dis 9: 154-156.

77. Uslan DZ, Jacobson KM, Kumar N, Berbari EF, Orenstein R (2006) A woman with fever and rash after African safari. Clin Infect Dis 43: 609, 661-609, 662.

78. Walker J (2000) ProMed-mail 20001107.1943.

79. Weinberg JR, Wright PA, Cook GC (1989) Tropical pyomyositis associated with Trypanosoma brucei rhodesiense infection in a Europid. Trans R Soc Trop Med Hyg 83: 77-79.

80. Zoller T (2008) personal communication.
